# Perfluorocarbon Liquid: Its Application in Vitreoretinal Surgery and Related Ocular Inflammation

**DOI:** 10.1155/2014/250323

**Published:** 2014-03-30

**Authors:** Qi Yu, Kun Liu, Li Su, Xin Xia, Xun Xu

**Affiliations:** ^1^Department of Ophthalmology, Shanghai First People's Hospital Affiliated to Shanghai Jiaotong University, No. 100 Haining Road, Shanghai 200080, China; ^2^Shanghai Key Laboratory of Fundus Diseases, Shanghai 200080, China

## Abstract

The application of perfluorocarbon liquids has been well acclaimed in vitreoretinal surgery. Its unique physical properties make it an ideal intraoperative tool to improve the efficiency and safety of surgical procedures in complicated cases. The main functions of perfluorocarbon liquids in vitreoretinal surgery include relocating and fixing the detached retina, displacing the subretinal and subchoroidal to fluid anteriorly, revealing proliferative vitreous retinopathy (PVR) for further maneuvers, protecting the macula from exposure to chemicals with potential toxicity, and assisting the removal of foreign body. The related clinical applications include retinal detachment with severe proliferative vitreoretinopathy, giant tear, diabetic retinopathy (DR), retinopathy of prematurity (ROP), and posterior dislocated crystalline and intraocular lenses. The application of perfluorocarbon liquids has been expended over the past fewer years. Several PFCLs related ocular inflammations have been observed in *in vitro* studies, animal studies, and clinical follow-up. The complete removal of PFCLs is recommended at the end of the surgery in most cases.

## 1. What Are Perfluorocarbon Liquids?

Perfluorocarbon liquids (PFCLs) are a serial of fluorochemicals in which all the hydrogen atoms are replaced by fluorine [1].

PFCLs are not found in nature. Those compounds are industrially produced by methods such as electrochemical fluorination, oligomerization, and telomerization [2].

Characteristically PFCLs have high specific gravity ranging from 1.76 to 2.03, low surface tension, and viscosity [3, 4]. These physical properties make perfluorocarbon liquids an ideal for intraoperative tool in vitreoretinal surgery.

## 2. Commonly Used Types of Perfluorocarbon Liquids and Their Characteristics

Several kinds of perfluorocarbon liquids have been applied in ophthalmology in different countries. They are perfluoro-octane (PFO), perfluoroperhydrophenanthrene (Vitreon), perfluorodecalin (PFD), perfluorotributylamide (PFTB) and perfluorooctylbromide (PFOB), and so forth [5, 6].

The physical properties of PFCLs including high specific gravity, moderate surface tension, low viscosity, and optical clarity and transparency make them ideal intraoperative tools for vitreoretinal surgery [7].

The gravity of the above-mentioned PFCLs ranges from 1.76 to 2.30, which empowers the liquid to flat the detached retina and displace the underneath fluids anteriorly [8]. The transparency of PLFCs as a colourless and clear media also ensures that the intraoperative usage of the fluid does not affect the observation of the operators during the surgery and intraoperative photocoagulation. The surface tension of PFCLs ensures the liquid staying relatively cohesive after been injected into the vitreous cavity [9, 10]. The low viscosity makes PFCLs easier to handle while injection and removal [11].

## 3. The History of PFCLs in Ophthalmology

The potential application of PFCLs in medicine was discovered by Clark Jr. and Gollan in 1966. Mammals including mice and cats in the containers filled with fluorocarbon managed to survive after weeks [12]. With further investigation, PFCLs' capacity to carry oxygen was confirmed and later developed as blood substitute [13]. In 1982, Haidt et al. used PFCLs as vitreous tamponade in experiments [14]. Zimmerman and Faris used PFCLs as intraoperative tool to relocate the detached retina in 1982 [15]. In 1987, after* in vivo* and* vitro* studies of the efficiency and safety of intraoperative application, Chang et al. use PFCLs in vitreous surgeries of retinal detachment patients with severe PVRs [16].

## 4. The Functions and Related Indications in Vitreoretinal Surgery

### 4.1. Relocating and Stabilizing the Detached Retina for Further Maneuvers

The gravity of PFCLs in use is about 2 times greater than perfusion solution. So while injected into the vitreous cavity during vitrectomy, the gravity of PFCLs generates a force against the interface downwards. While it is against the detached retina, the injected PFCLs relocate and immobilize the detached posterior retina. And, while PFCLs are gradually injected into the vitreous cavity, the subretinal fluid is pushed anteriorly and thus into the vitreous cavity through the retinal breaks, which often results in avoiding retinotomy for posterior drainage [17]. In some cases, this process can provide information about the location of the unidentified peripheral breaks if subretinal fluid drainage is observed through breaks other than the identified retinal breaks.

#### 4.1.1. Retinal Detachment with Severe PVR

The very first application of PFCLs in vitreoretinal surgery was in patients of retinal detachment with severe PVR [18]. The application of PFCLs has changed surgical management of PVR. Before that, anterior PVR dissection was performed first and then followed by dissection of posterior PVR. The use of PFC eyes with retinal detachments complicated by PVR permits initial dissection of posterior PVR. The injection of PFCLs after initial dissection aids in opening the funnel to provide better visualization of proliferative membranes and a more thorough removal of the membranes [19].

Regarding the retinal reproliferation after surgery, Greve et al. reported that the intraoperative use of PFCLs in vitreoretinal surgery does not prevent postoperative surgery reproliferation, but it does reduce the severity since the application of PFCLs allows for a more complete removal of the epiretinal membranes. Several other studies have demonstrated the usefulness of PFCLs as an intraoperative tool, diagnostically and therapeutically as well in patients with retinal detachment and PVR [20].

#### 4.1.2. Giant Tears

Retinal detachment with giant tears has been a challenging field in vitreoretinal surgery. The mobility of the detached retina is relatively higher and more difficult to be manipulated due to the size and location of the retinal tears. The application of perfluorocarbon liquids stabilizes the detached retina during vitrectomy and displaces the subretinal fluid [21]. Vitreon and perfluorooctane were well studied for clinical use [22, 23].

Zhioua et al. evaluated the usefulness of an intraoperative injection of PFCLs in 17 eyes with giant retinal tears (between 90 and 220 degrees) associated with grade 3 PVR. They found an improvement in both anatomical and functional prognoses [24].

In the perfluorooctane study group's work in 2002, Scott and colleagues included 212 eyes of 212 patients with giant tears and followed a median of 3.5 months. 59% percent of the postoperative visual acuity was improved, 24% remained stable, and 16% was worsened. At 6 months, the retina reattachment rate was 76% [25].

With the intraoperative use of PFCLs, giant tears with no severe PVR, the chance of preserving the lens during surgery has been increased. In some cases, no tamponade of silicon oil is needed [26, 27].

#### 4.1.3. Diabetic Retinopathy

Application of PFCL during vitreous surgery for proliferative diabetic retinopathy (PDR) has been reported by several authors, mostly in quite severe cases. The functions of PFCLs during surgery are similar to cases of retinal detachment with severe PVR. PFCL is a useful adjunct during vitrectomy for severe PDR, especially to flatten shrunken retina. PFCL is also efficient to flatten retinal detachments that appeared when relieving tight vitreoretinal adhesion [28]. The application of PFCL also provides a better condition to perform panretinal photocoagulation if needed with lower energy.

In the study of Imamura and his colleagues, the surgical results were acceptable although the follow-up time was relatively short. PFCL was used in the most complicated cases among PDR patients. In those 18 cases, they all showed macular tractional detachment; two had combined rhegmatogenous retinal detachment and one had PVR due to a previous failed vitrectomy for PDR. The anatomical success rate was 89%, and a visual improvement was found in 10 eyes (55%) [29].

#### 4.1.4. Retinopathy of Prematurity and Other Complex Pediatric Retinal Detachment

Perfluorooctane has been used in complex pediatric retinal detachment with severe PVR. While the posterior proliferative changes were in the inferior retina and gas or silicone was considered less effective or ineffective, perfluorooctane can be considered as a temporary postoperative tamponade [30, 31].

### 4.2. Floating the Foreign Bodies in the Vitreous Body

A confirmed vitreous foreign body often requires surgical removal. For nonmetal foreign bodies with gravity less than PFCLs (1.76–2.03), the injection of PFCLs into the vitreous body before removal can lift the foreign body away from the retina, thus simplifying the procedures of removal and, mostly, improving the safety of the process [32, 33].

#### 4.2.1. Penetrating Trauma with Posterior Foreign Body

For patients with posterior foreign body after penetrating trauma, PFCLs can assist the removal of light foreign bodies. Trauma cases are often complicated by lens injury, retinal detachment, and vitreous and subretinal or choroidal hemorrhage. The application of PFCLs can also help with the management of retinal detachment, hemorrhage, and proliferation [34].

#### 4.2.2. Posterior Dislocated Crystalline and Intraocular Lenses

In cases of posterior dislocated crystalline or intraocular lenses (IOLs), PFCLs can be applied for the same reason for low gravity vitreous foreign bodies. Even when the dropped lens coexists with a retinal detachment, PFCLs are valuable intraoperative tools because they can aid in the removal of fragments from the vitreous cavity without eliciting iatrogenic retinal damage. Similarly, removing a dislocated IOL by injecting PFCL reduces the risk of injuring the retina during the maneuvers required to retrieve an IOL from the posterior vitreous cavity [35, 36].

Jang reported a modified technique of phacoemulsification in dislocated crystalline lenses in a study of 11 eyes [37]. After the vitreous and posterior hyaloid membranes were removed, perfluorocarbon liquid (PFCL) was injected at the posterior pole to fill the vitreous cavity. The dislocated lens floated on the PFCL, and the injection was ceased once the lens had risen to the iris plane. The lens was then removed from the anterior chamber using standard phacoemulsification procedures. During the phacoemulsification, the PFCL provides a support underneath the nucleus, like a trampoline, and even small fragments can be completely removed. The best-corrected visual outcome was reported more than 20/40 in 11 eyes.

### 4.3. Protecting the Macula

During vitrectomy, for the purpose of treatment or assisting a procedure, biochemically active agents or drugs may be injected into the vitreous cavity to avoid related toxic effects to the macula. Before the injection of the potentially toxic agents, a small amount of PFCLs is injected into the vitreous cavity to form a bubble covering the macula area, thus separating the macula from the potentially toxic agents [38–40].

### 4.4. Suprachoroidal Hemorrhage

A suprachoroidal hemorrhage is defined as a hemorrhage in the suprachoroidal space. PFCLs may be useful for expressing suprachoroidal hemorrhage from sclerotomies after vitrectomy. When injected directly over the retina, PFCLs create a posterior tamponade effect, unlike air or gas, by pushing the subchoroidal hemorrhage anteriorly and making it exit through the anterior sclerotomies [41, 42].

PFD or PFO has also been successfully used as an endotamponade tool combined with a tissue plasminogen activator for the treatment of subfoveal hemorrhages in cases of exudative age-related macular degeneration ARMD by preventing or reducing the risk of massive subretinal hemorrhages as a possible complication of treating exudative ARMD [43, 44].

## 5. The Time-Related Ocular Inflammations Caused by PFCLs

### 5.1. *In Vitro* Studies and Animal Studies

Previous* in vitro* studies have evaluated the effects of direct toxicity and damage due to PFO gravity on human retinal pigment epithelium cells and retinal ganglion cells. They found that PFO was toxic to the ARPE-19, a spontaneously arising human RPE cell line after 7 days of exposure, and PFO generated damage through the mechanical force imparted to retinal ganglion cells [45, 46].

The short tamponade of PFCLs has been reported relatively safe by animal studies. Electron-microscopic studies of intravitreal perfluorotributylamine, perfluorodecalin, and perfluorooctane placed in pig eyes for up to 3 hours indicate a lack of retinal changes. Electrophysiological studies and morphological examination using both light and electron microscopy have revealed no evidence of retinal toxicity after perfluorophenanthrene intravitreal placement for periods up to 48 hours [47, 48].

Intraretinal macrophages and foam cells are observed after 1 to 2 weeks intravitreal placement of PFO, PFD, and PFOB [49]. The narrowing of the outer plexiform layer and degenerative thinning of the outer nuclear layer can be observed progressively as the perfluorocarbon liquids remain longer within the eye [50]. Only the inferior retina exhibits these changes, which are believed to result from the mechanical effects of the high specific gravity of PFCLs which exerting prolonged pressure against the retina. These changes may be only species specific, and similar observations have been made of the superior retina of rabbit eyes following silicone oil injection. Small droplets of perfluorooctane (0.1 mL) injected into the rabbit vitreous appear to be well tolerated, eliciting a macrophage response but no retinal alteration at 6 months [51, 52].

The purification and chemical stability of the perfluorocarbon liquids are highly related to PFCLs induced ocular inflammation. During the production of PFCLs, the impurities are often partly fluorinated substances H and double bonds which are biochemical active and result in ocular inflammation and cytotoxicity [53].

Velikay et al. reported the clinical and histological observations of PFD and PFOB both extremely purified and with 5–30% impurities as short time tamponade in a period of 8 weeks. PFD and PFOB which were not purified caused severe inflammation and retinal detachment. Distinct disarrangement of the outer nuclear layer, vacuolization in the inner nuclear layer, and both edema and vacuolization of the ganglion cell layer were observed [54].

### 5.2. Clinical Observation

PLFCs related ocular inflammation is also observed in clinical settings especially in cases of subretinal PFCLs [55]. Significant pigment epithelial atrophy throughout the area was vacated by subretinal PFCLs [56]. Subretinal PFCL also can cause local functional changes in the sensitivity of the retina, which has been demonstrated by SLO microperimetry.

Young patients are probably at higher risk for developing severe inflammation. Eyes that develop persistent inflammation, secondary membrane formation, or recurrent RD will need reoperation for removal of this material [57–59].

The use of PFO has been reported to be involved in the occurrence of sticky silicone oil. Interactions of PFCL with silicone oil or heavy silicone oil and variations in temperature are responsible for the increase in shear viscosity and opacity of the oil [60]. PFCL interacts with silicone, dissolving small amounts of the oil into solution over time. And the surface tension of the surrounding aqueous material and/or contamination of silicone oil with PFCL reduced. While the presence of silicone oil remnants on the retina did not cause lasting side effect, forceful attempts at removal can lead to complications [53].

## 6. Recent Developments in PFCLs

### 6.1. Perfluorocarbon-Perfused Vitrectomy

One of the recent developed applications of PFCLs in vitrectomy is perfluorocarbon-perfused vitrectomy. This system employs PFCL perfusion instead of balanced salt solutions (BSSs) during vitrectomy [61]. Oxygenated or nonoxygenated PFCL is used in a recycling or a nonrecycling system for this procedure. PFCL-perfused vitrectomy benefits from several properties of PFCLs [62–65].

In cases with severe diabetic retinopathy, oxygenated PFCLs can be an advantage for the circulation compromised retina. The high oxygenated PFCLs also allow the vitreous surgeons to raise the intraocular pressure to a relatively higher level during a hemostatic surgery. The immiscibility of PFCLs with blood and other intraocular fluids also allows for visible vitreous removal. And most of the functions of PFCLs as intraoperative tool are also applicable while being used as perfusate, such as relocating and stabilizing the detached retina for further maneuvers [66].

### 6.2. Hydrogenated Hydrofluorocarbon Liquids (HFCLs)

To improve the intraocular tolerance of PFCLs as vitreous tamponade, modifications have been made to reduce the specific gravity of the liquids [67]. Hydrogenated hydrofluorocarbon liquids (HFCLs) were developed with reduced specific gravity values and increased lipophilic properties. HFCLs, such as F6H6, F6H8, O44, and O62, were developed to substitute for PFCLs as long-term tamponade in vitreoretinal surgery [68–71]. Due to their properties, these compounds have the potential to remove intraocular silicon oil remnants [72, 73].

## 7. Conclusion

Regarding the unique physical properties of PFCLs, they have been well accepted as intraoperative tools for vitreoretinal surgery. The application of PFCLs has simplified the surgical procedures and improved the safety of the process especially in severe and complicated cases. Continuous efforts will be made to further improve the compatibility and reduce the related toxicity and ocular inflammation.
